# A survey dataset to better understand the honey bee industry, use and value of natural resources and challenges for beekeepers in Western Australia: A beekeepers’ perspective

**DOI:** 10.1016/j.dib.2022.108639

**Published:** 2022-09-25

**Authors:** Cheryl Day, Benedict White

**Affiliations:** UWA School of Agriculture and Environment, the University of Western Australia, M087/35 Stirling Highway, Crawley, Western Australia 6009, Australia

**Keywords:** Apiary site, Native bush resources, Honey bee flora, *Apis mellifera*, Ecosystem services, Pollination services, Survey data, Beekeeping

## Abstract

The summary data presented in this paper describes beekeeping practices, use of natural resources and economic attributes associated with honey bee products, native flora and environmental challenges relating to apiary sites. Despite being a well-established industry, information and data about productivity and the behavior of beekeepers, particularly those who migrate across the state of Western Australia, is lacking. We developed an online quantitative survey, the Natural Resources for Beekeepers Questionnaire (Western Australia) 2020-21, the first comprehensive, spatially referenced survey of beekeepers in Western Australia since 1990. It is also the first survey of small-scale amateur beekeepers that estimates their supply to the local honey market. For commercial beekeepers, a focus of the survey was to estimate the value of apiary sites and the productivity of migratory beekeepers. The data gives measures related to the production system and profitability of the Western Australian beekeeping industry, focusing on the 2019-2020 season and historical production. It includes tables describing memberships and certification; years beekeeping; hive types; apiary site availability, productivity, use, exchange and value; logistics; pollination services; honey bee products, sales and distribution; yields by season and site; targeted flora and commercial significance; recovery after bush fire and logging; labour details; operating costs; and asset values. The dataset in this paper is a subset of the survey results as aggregated summary statistics, categorized by type of beekeeper (Backyard, Hobbyist-Amateur and Commercial) and across eight regions (IBRA7 - Interim Biogeographic Regionalization for Australia). The online survey questionnaire is provided with this paper. Access to the survey offers the opportunity for reproducibility of a complex online questionnaire in the future and/or for other regions. This dataset will allow a more comprehensive assessment of the implications of natural resource management decisions in the future and the potential for strategic development of the beekeeping industry.

**Specifications Table**

**Table:** 

Subject	Agricultural Sciences - Agricultural Economics
Specific subject area	Honey bee (A*pis mellifera*) beekeeping and apiary sites, Valuing natural resources, Sustainable industry and Ecosystem services, Natural resource management decisions, Honey bee products
Type of data	Tables and Figures
How the data were acquired	Online survey questionnaire “*Natural Resources for Beekeepers Questionnaire (Western Australia) 2020-21*“ developed using Qualtrics software. Survey questionnaire available at 10.26182/j335-5867 The target population for the survey included all current and retired beekeepers in Western Australia in December 2020 through to June 2021. In addition, non-beekeepers who held a licence for registered apiary sites on land managed by the state government's Department of Biodiversity, Conservation and Attractions (DBCA) were also included. This target population could be stratified into industry sub-groups such as Backyard beekeepers; Hobbyist-Amateur (part-time) beekeepers; Commercial (full-time) beekeepers; Retired commercial beekeepers; and Non-beekeepers with registered apiary sites. Privacy and confidentiality restrictions meant it was not possible to draw a random stratified sample from the target population directly. Therefore, those who completed the questionnaire were self-selected, however this still provided a sample from a cross section of the whole population which reflected the relative frequency of each strata. Every effort was made to invite all potential participants to complete the survey voluntarily and no payment or incentive was offered. DBCA shared the email addresses of all beekeepers who held a licence for a registered apiary site, and we sent an email inviting them to participate in the survey online. In Western Australia, registration with the Department of Primary Industries and Regional Development (DPIRD) is mandatory. DPIRD sent an email to all registered beekeepers inviting them to participate with a link to the online survey and a reminder follow up email. For privacy reasons these email addresses could not be shared with us. To increase the response rate, promotion of the survey and link were also delivered through industry networks, such as the Beekeeping Industry Council of Western Australia and the Western Australian Apiarist Society, targeting both commercial and hobbyist beekeepers. Communication via newsletters, websites and Facebook were also used, as well as reminder emails.
	The questionnaire was developed to meet our research project purpose, to value bush apiary sites in the south west of Western Australia and provide spatially referenced economic and biophysical data. The online survey questionnaire design is a novel approach to gathering data from a diverse industry. Respondents’ answered questions tailored to their scale of production and use of natural resources with the pathway through the questionnaire dependent on previous responses. This avoided the presentation of irrelevant questions and allowed a single questionnaire to be used for small-scale backyard beekeepers and large scale migratory commercial beekeepers.
Data format	Raw – for peer review process only (Confidential Restricted) Cleaned and Analyzed
Description of data collection	Data was collected between December 2020 and June 2021 through an online survey developed in Qualtrics. Potential participants from our target population were invited to complete the online survey anonymously using their personal electronic devices such as mobile phone, iPad or personal computer by following the survey link. Respondents could suspend the questionnaire and return to the link using the same computer and web browser when convenient. Response data underwent a thorough data cleaning and validation process using Stata/SE 16.1 [6] to exclude irrelevant data and duplicates, fix errors and missing data, identify outliers and verify data accuracy. The data was stratified according to beekeeper classifications. If a respondent failed to provide the number of sites and number of hives, or the amount of honey harvested for the study season 2019-20 they were categorized as invalid and removed from the data.
Data source location	City/Town/Region: Western Australia Country: Australia
Data accessibility	Repository name: Pure, The University of Western Australia Research Repository Data identification number: 10.26182/j335-5867 Direct URL to data: https://research-repository.uwa.edu.au/en/datasets/a-survey-dataset-to-better-understand-the-honey-bee-industry-use- Raw data for peer-review process only – Confidential Restricted

## Value of the Data

• Valuing natural resources for beekeepers and identifying the impact of management decisions on bee health.
• This data set provides useful information about operating as a beekeeper at various levels of commercial and non-commercial operation, use and value of natural resources at an apiary site and challenges facing the industry with the loss of nectar and pollen sources.
• This data set fills a gap in knowledge about the production of honey bee products and pollination services, particularly for non-commercial and migratory beekeepers.
• The data set may be used by other researchers, government agencies, industry bodies and individuals as a data source, further analysis and/or evidence for policy and decision-making. The accompanying questionnaire may be used by others who aim to repeat it to compare over years or reproduce partially or wholly in other jurisdictions.
• The authors’ work provides extensive detail for a cross section of beekeepers, including capturing the migratory behaviour to source nectar and pollen, and the potential for industry benchmarking, hindsight assessments and strategic planning for the future.

## 1 Data Description

The dataset file contains sixty-six tables of analyzed and aggregated survey data (Dataset link) [1]. Here tables are presented in a non-identifiable format to ensure anonymity. Each table refers to a survey question or combination of survey questions as described. A summary of descriptive statistics, sample size and reference to survey question number(s) are provided in tabulated format in Excel. There are eighteen Excel worksheets within the workbook, which comprise of tables relating to similar topics (Table 1).

**Table 1:** Content in dataset file for the natural resources for beekeepers questionnaire (Western Australia) 2020-21 survey results.

Worksheet			
**1**	**Beekeeper sample**	**10**	**Pollination**
	Survey sample		Pollination services
**2**	**Summary**	**11**	**Regional site summary**
	Key production parameters of the Western Australian apiculture industry		Regional site summary
**3**	**Membership and Certification**	**12**	**Honey production by region**
	Industry memberships and certifications		Honey harvest by region
**4**	**Year started**	**13**	**Yields by season**
	Year started beekeeping, by beekeeper category, in five-year intervals		Honey yields by type of season
**5**	**Max hive number**	**14**	**Yields by site**
	Largest number of hives managed in any year, including nucleus hives, by beekeeper category		Honey yields by site description
**6**	**Hive type**	**15**	**Region profile - site specifics**
	Types of hives used, by beekeeper category		Region profile, site specific use and exchange
**7**	**Site option**	**16**	**Region profile - flora**
	Type and number of sites available for use		Region profile, targeted flora and commercial significance
**8**	**Logistics**	**17**	**Extract, distribute & sell**
	Hive site shifts, fleet vehicles and distances travelled		Extraction, distribution and sales
**9**	**Homebase**	**18**	**Labour, costs & assets**
	Beekeepers' homebase, backyard hive site locality and honey harvested		Labour, operating costs and asset values

The results given below in this paper are a selection of tables to provide a snapshot of the beekeeping industry of Western Australia, and a sample of the full dataset.

Due to human research ethics requirements and agreement with the Beekeeping Industry Council of Western Australia and conditions in the respondents’ consent, responses must be non-identifiable and aggregated, therefore raw survey data cannot be made publicly available.

**Table 2:** Survey sample size, by beekeeper category and whether questionnaire was completed or not.

	Beekeeper category				
Questionnaire status	Backyard	Hobbyist-Amateur	Commercial	Invalid	Total
Incomplete	23	84	29	100	236
Completed	162	244	25	51	482

**Total**	**185**	**328**	**54**	**151**	**718**

Survey questions and answer options as seen by respondent can be viewed in the survey questionnaire (Survey link).

### 1.1 Sample Description (Table 2 and Fig. 1)

There were approximately 4000 beekeepers registered in Western Australia at the time the survey was opened. Including invalid responses there was an 18% response rate, however when invalid responses were excluded the response rate was about 14% of the population.

**Fig. 1:**
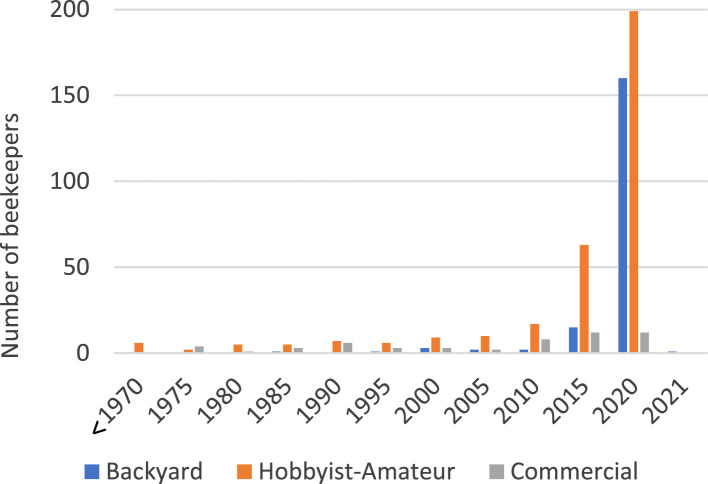
Years of experience beekeeping by respondents, year started beekeeping, by beekeeper category, in five-year intervals [Q4].

Beekeepers were reclassified into a beekeeper category based on number of hives, number and type of sites and honey harvested, as described below.

• Backyard beekeeper - anyone only using backyard sites; and has 2 or less hives; and 5 or less sites; and not missing harvested honey 2019-20 season amount.
• Hobbyist-Amateur beekeeper – anyone who does not meet Backyard beekeeper criteria; and has 39 or less hives; and any number of sites; and not missing harvested honey 2019-20 season amount.
• Commercial beekeeper – anyone who does not meet Backyard beekeeper or Hobbyist-Amateur beekeeper criteria; and has 40 or more hives; and any number of sites and not missing harvested honey 2019-20 season amount.

Any respondents with missing values for the number of sites; and number of hives; or honey harvested for 2019-20 season were categorized as invalid entries.

### 1.2 Key production parameters of the Western Australian apiculture industry (Table 3)

Production values, sales and distribution, and sites used relate to beekeeping season 1 July 2019–30 June 2020, inclusive of incomplete responses.

**Table 3:** Key production parameter summary, by beekeeper category [Various questions, repeated in detail in other tables in dataset file].

*Backyard beekeepers*						

Response	Obs	Mean	Std Dev.	25th PC	50th PC	75th PC

Number of hives	185	1	0.5	1	1	2
Honey per hive (kg/hive)^a^	143	24	22.1	10	20	30
Honey from pollination services (kg)	0					
Honey harvested, including pollination (kg)^a^	143	32	29.3	10	25	40
No honey harvested, including pollination (kg)^b^	42					
Honey sold/distributed (kg)	117	32	28.6	12	24	40
Pollen sold/distributed (kg)	2	11	13.4	1	11	20
Wax sold/distributed (kg)	7	2	1.5	1	2	3
Propolis sold/distributed (kg)	0					
Royal jelly sold/distributed (kg)	0					
Queens sold/distributed (no.)	0					
Nucleus hives sold/distributed (no.)	3	2	1.7	1	1	4
Packaged bees sold/distributed (no.)	0					
More than one site used (no.)	9	1	0.4	1	1	1
Operating costs - not required	0					
Number of hives by 2025	155	3	3.8	1	2	2

*Hobbyist-Amateur beekeepers*						

Response	Obs	Mean	Std Dev.	25th PC	50th PC	75th PC

Number of hives	328	8	7.8	3	5	11
Honey per hive (kg/hive)^a^	267	19	23.8	6	13	25
Honey from pollination services (kg)	0					
Honey harvested, including pollination (kg)^a^	272	146	229.7	20	60	175
No honey harvested, including pollination (kg)^b^	20					
Honey sold/distributed (kg)^a^	240	148	240.1	20	58	183
Pollen sold/distributed (kg)^c^	n/a					
Wax sold/distributed (kg)	48	12	18.0	3	5	15
Propolis sold/distributed (kg)	3	2	2.8	0	0	5
Royal jelly sold/distributed (kg)^c^	n/a					
Queens sold/distributed (no.)	3	17	11.0	10	12	30
Nucleus hives sold/distributed (no.)	21	7	9.0	2	4	10
Packaged bees sold/distributed (no.)	0					
Sites used (no.)	240	2	2.1	1	2	3
Operating costs per hive ($/yr)	37	190	179.0	75	142	229
Number of hives by 2025	230	15	37.3	3	5	12

*Commercial beekeepers*						

Number of hives	54	275	320.5	68	139	336
Honey per hive (kg/hive)^d^	33	46	40.5	19	38	56
Honey from pollination services (kg)	5	880	938.5	150	350	1,800
Honey harvested, including pollination (kg)^a^	37	16,156	36,587.9	1,530	3,400	13,800
Honey sold/distributed (kg)^e^	30	9,557	12,981.3	1,300	3,150	15,000
Pollen sold/distributed (kg)^e^	7	2,994	3,862.5	57	500	5,194
Wax sold/distributed (kg)^e^	18	785	1,400.4	30	86	600
Propolis sold/distributed (kg)^e^	2	51	69.3	2	51	100
Royal jelly sold/distributed (kg)^c^^e^	n/a					
Queens sold/distributed (no.)^e^	5	183	149.8	100	200	200
Nucleus hives sold/distributed (no.)^e^	10	28	31.8	10	11	50
Packaged bees sold/distributed (no.)^c^	n/a					
Sites used (no.)	42	12	13.4	5	9	13
Operating costs per hive ($/yr)	27	403	426.0	60	250	674
Number of hives by 2025	22	488	566.3	90	335	500

Notes

25th PC i.e. 25th percentile, 25% of values are below this value.

50th PC i.e. 50th percentile, 50% of values are below this value.

75th PC i.e. 75th percentile, 75% of values are below this value.

a Excludes values < 1

b Includes values < 1

c - n/a - Not available for publication, too few observations

d Excludes values < 5

e Excludes values < = 1

### 1.3 Apiary Site Types, Regional Location and Usage Purpose (Table 4 and Figs. 2 and 3)

There are different types of apiary sites used by beekeepers. We have described apiary sites as follows.

• Backyard site in an urban environment – sites in a populated area with parks and garden flora the primary food source.
• Registered site from DBCA – sites in National and conservation parks, state forests, nature and timber reserves, pastoral leases, mining tenements and unallocated crown land where the government Department of Biodiversity, Conservation and Attractions manage apiary permits and licences.
• Private site – any privately owned rural land used for honey production or bee health, not specifically for pollination.
• Other – roadsides, council/shire land etc.
• Pollination sites – sites in agricultural fields for the purpose of pollination services, where honey bees interact with food and fibre plants at the onset of crop flowering to produce seed, nut, fruit and vegetables, and not for the specific purpose of honey production or bee health.

**Table 4:** Type and number of apiary sites available for use by the beekeeper in the beekeeping season 1 July 2019–30 June 2020, by beekeeper category [Q44].

Site type	Observations	Mean	Std Dev.	25th PC	50th PC	75th PC
*Backyard beekeepers*						
Backyard in an urban environment	185	1	0	1	1	1
Registered (DBCA) - own	0					
Registered (DBCA) - other people's	0					
Private	0					
Other	0					
Pollination	0					
**Response total**	**185**					
*Hobbyist-Amateur beekeepers*						
Backyard in an urban environment	169	2	2	1	1	3
Registered (DBCA) - own	7	1	0	1	1	1
Registered (DBCA) - other people's	2	13	10	6	13	20
Private	209	3	5	1	1	3
Other	3	2	1	1	1	3
Pollination	6	2	1	1	1	3
**Response total**	**328**					
*Commercial beekeepers*						
Backyard in an urban environment	11	4	3	1	4	8
Registered (DBCA) - own	25	80	123	11	56	100
Registered (DBCA) - other people's	17	78	179	5	7	26
Private	53	29	51	5	10	32
Other	3	14	20	2	3	37
Pollination	18	12	39	1	3	5
**Response total**	**54**					

Notes

25th PC i.e. 25th percentile, 25% of values are below this value.

50th PC i.e. 50th percentile, 50% of values are below this value.

75th PC i.e. 75th percentile, 75% of values are below this value.

Response total is the number of respondents who answered this question.

**Fig. 2:**
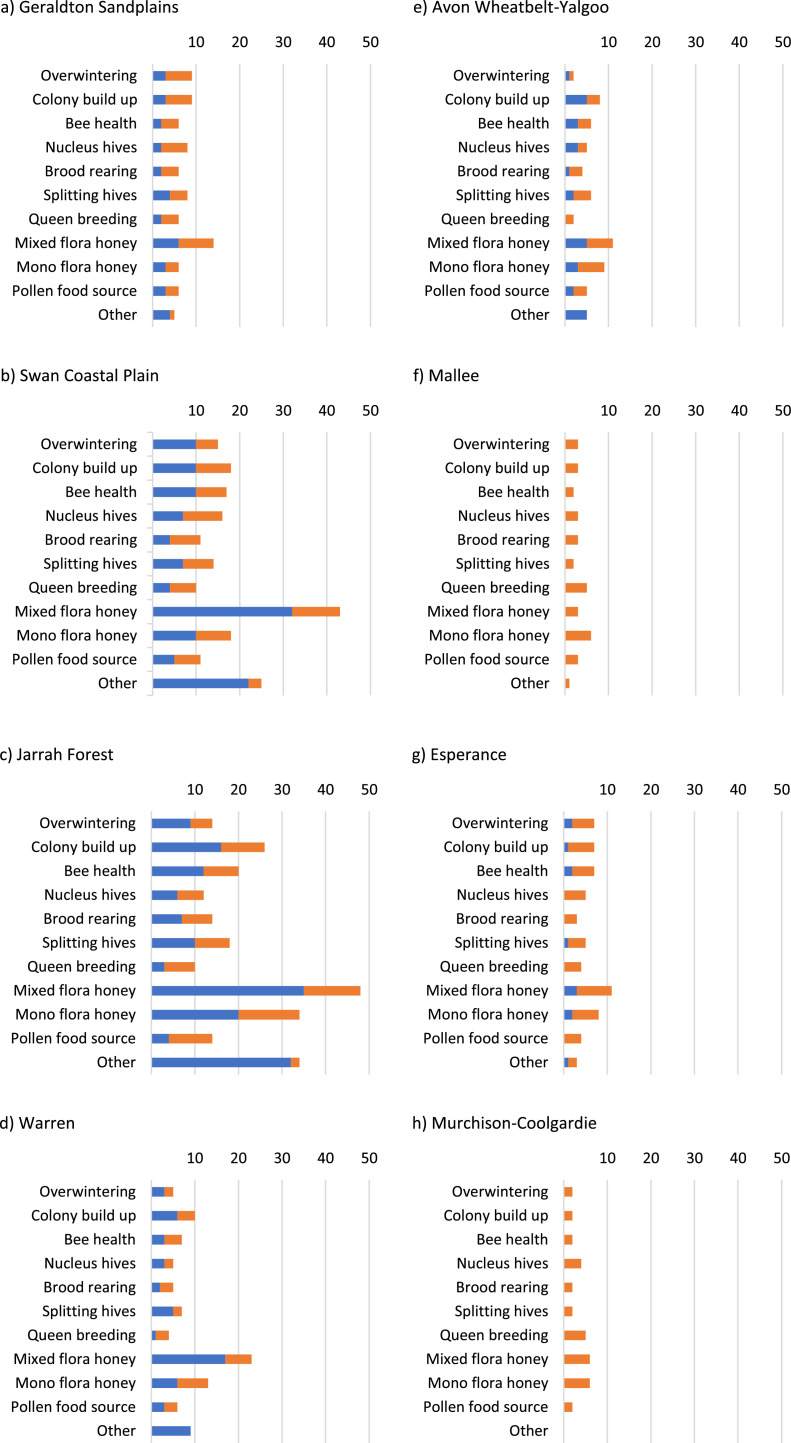
Purpose of using sites in each region of the south west, by beekeeper category. Blue: Hobbyist-Amateur beekeepers, Orange: Commercial beekeepers [Q126].

**Fig. 3:**
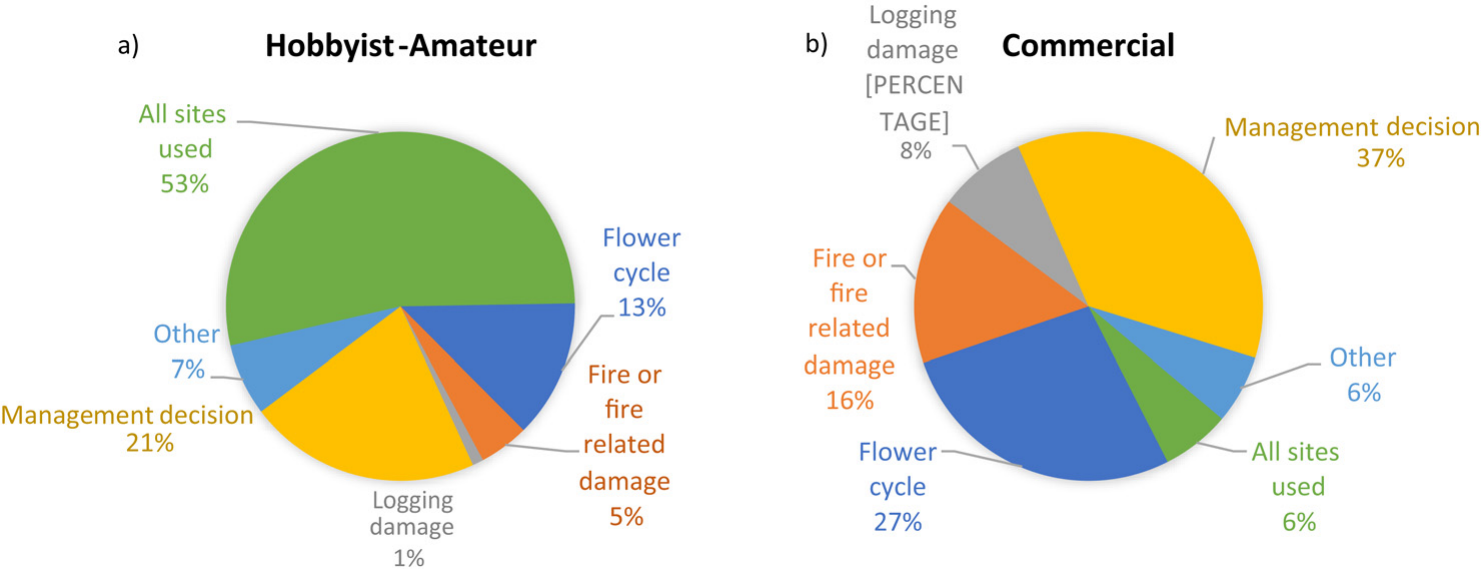
Reasons why not all sites used in a year, by beekeeper category (a) Hobbyist-Amateur, (b) Commercial [Q76].

### 1.4 Honey Production and Yields by Region (Table 5, Table 6, Table 7)

**Table 5:** Honey harvested in season July 2019-June 2020, by beekeeper category and by site region [Q93, Q94, Q95].

	Honey harvested (kg/yr)						Pollination crop honey incl.
	Observations	Mean	Std Dev.	25th PC	50th PC	75th PC	Observations
*Backyard beekeepers*^a^^,^^b^							
Geraldton Sandplains	0						0
Swan Coastal Plain	5	58	50.6	40	45	60	0
Jarrah Forest	1	20		20	20	20	0
Warren	0						0
Avon Wheatbelt-Yalgoo	0						0
Mallee	0						0
Esperance	0						0
Murchison-Coolgardie	0						0
Northwest-Nullarbor-Deserts	0						0
**Response total**	**6**						**0**
*Hobbyist-Amateur*^a^							
Geraldton Sandplains	9	331	374.6	14	250	700	0
Swan Coastal Plain	95	165	248.1	26	100	200	1
Jarrah Forest	74	138	246.0	20	40	150	1
Warren	31	106	116.9	35	80	160	0
Avon Wheatbelt-Yalgoo	9	51	66.6	10	20	50	0
Mallee	1	200		200	200	200	0
Esperance	5	50	37.8	11	60	80	0
Murchison-Coolgardie	0						0
Northwest-Nullarbor-Deserts	1	60		60	60	60	0
**Response total**	**209**						**4**
*Commercial*^c^							
Geraldton Sandplains	8	9,389	20,677.5	215	425	6,900	0
Swan Coastal Plain	16	7,790	16,162.4	200	2,000	3,000	0
Jarrah Forest	19	9,785	29,374.2	250	2,000	7,000	0
Warren	4	975	1,231.2	300	500	1,650	0
Avon Wheatbelt-Yalgoo	5	4,401	7,129.8	700	1,300	3,000	0
Mallee	2	3,050	3,606.2	500	3,050	5,600	0
Esperance	5	11,080	9,478.0	2,000	13,000	20,000	0
Murchison-Coolgardie	4	5,205	9,864.7	160	350	10,250	0
Northwest-Nullarbor-Deserts	0						0
**Response total**	**31**						**1**

Notes

25th PC i.e. 25th percentile, 25% of values are below this value.

50th PC i.e. 50th percentile, 50% of values are below this value.

75th PC i.e. 75th percentile, 75% of values are below this value.

a Excludes honey harvested values < 1

b Backyard beekeepers with more than 1 backyard site.

c Excludes honey harvested values < = 1 Response total is the number of respondents who answered the question.

**Table 6:** Estimated honey yield per hive, based on honey harvested in season 2019-2020 and number of hives used in each region, by beekeeper category, and by region.

	Estimated hive honey yield (kg/hive)^c^					
	Observations	Mean	Std Dev.	25th PC	50th PC	75th PC
*Hobbyist-Amateur*^a^						
Geraldton Sandplains	6	28	19.9	9	30	42
Swan Coastal Plain	59	22	24.9	6	14	28
Jarrah Forest	70	18	12.9	8	13	24
Warren	26	18	11.3	8	19	25
Avon Wheatbelt-Yalgoo	8	17	11.3	10	13	27
Mallee	0					
Esperance	5	21	15.8	10	11	30
Murchison-Coolgardie	0					
Northwest-Nullarbor-Deserts	1	33		33	33	33
**Response total**	**163**					
*Commercial*^b^						
Geraldton Sandplains	7	20	17.9	6	15	30
Swan Coastal Plain	15	41	54.4	13	27	42
Jarrah Forest	18	37	32.5	13	30	47
Warren	4	8	4.7	4	8	12
Avon Wheatbelt-Yalgoo	4	33	27.5	9	32	57
Mallee	2	15	9.7	8	15	22
Esperance	5	39	23.0	31	40	50
Murchison-Coolgardie	3	13	15.2	2	7	31
Northwest-Nullarbor-Deserts	0					
**Response total**	**29**					

Notes

25th PC i.e. 25th percentile, 25% of values are below this value.

50th PC i.e. 50th percentile, 50% of values are below this value.

75th PC i.e. 75th percentile, 75% of values are below this value.

a Excludes honey harvested values < 1

b Excludes honey harvested values <=1

c Excludes estimated yields <1 Response total is the number of respondents who answered the question.

**Table 7:** Estimated honey yield per site, based on honey harvested in season 2019-2020 and number of sites used in each region, by beekeeper category, and by region.

	Estimated site honey yield (kg/site)^c^					
	Observations	Mean	Std Dev.	25th PC	50th PC	75th PC
*Hobbyist-Amateur*^a^						
Geraldton Sandplains	6	173	157.0	14	167	333
Swan Coastal Plain	60	66	79.5	16	33	100
Jarrah Forest	70	87	150.4	20	34	120
Warren	28	69	114.3	20	37	66
Avon Wheatbelt-Yalgoo	9	31	32.6	10	20	50
Mallee	0					
Esperance	5	37	36.5	11	20	60
Murchison-Coolgardie	0					
Northwest-Nullarbor-Deserts	1	350		350	350	350
**Response total**	**167**					
*Commercial*^b^						
Geraldton Sandplains	8	1,247	1,630.4	88	265	2,633
Swan Coastal Plain	16	931	1,081.0	91	447	1,750
Jarrah Forest	19	1,334	1,511.8	125	305	2,667
Warren	4	333	322.7	66	313	600
Avon Wheatbelt-Yalgoo	4	1,600	1,224.7	850	1,150	2,350
Mallee	2	1,525	1,803.1	250	1,525	2,800
Esperance	5	2,784	4,096.8	400	1,300	2,000
Murchison-Coolgardie	4	934	1,600.2	102	160	1,767
Northwest-Nullarbor-Deserts	0					
**Response total**	**31**					

Notes

25th PC i.e. 25th percentile, 25% of values are below this value.

50th PC i.e. 50th percentile, 50% of values are below this value.

75th PC i.e. 75th percentile, 75% of values are below this value.

a Excludes honey harvested values < 1.

b Excludes honey harvested values < = 1.

c Excludes estimated yields <1 Estimated honey yield per site is based on honey harvested and number of sites used in each region [Q75]. Response total is the number of respondents who answered the question.

### 1.5 Natural Resources Utilized Including, Bee Flora Targeted, and Commercial Significance

Refer to dataset Table 16.2 Flora species or type of honey targeted by beekeeper and bees for each region, by specific flora species or simplified vegetation zones, by beekeeper category. [Q128, Q129 ] Dataset link.

The species/flora honey type ranking according to the commercial significance to the beekeeping operation is given as.

High: critical to have access to sites with this species to be commercially viable.

Medium: significant risk to commercial viability without access to sites with this species.

Low: without access to sites with this species commercial viability can continue with some impact.

### 1.6 Natural Resource Management Impact, Production After Fire and Logging (Table 8, Table 9, Table 10, Table 11)

**Table 8:** Ever had site and surrounding 3 km impacted by fire, by beekeeper category, by region [Q83].

	Fire impacted site			
Response	Yes	Unsure	No	Response total
*Hobbyist-Amateur*				194
Geraldton Sandplains	2	0	7	
Swan Coastal Plain	14	3	56	
Jarrah Forest	30	3	55	
Warren	7	2	26	
Avon Wheatbelt-Yalgoo	4	1	8	
Mallee	0	0	0	
Esperance	2	1	3	
Murchison-Coolgardie	0	0	0	
Northwest-Nullarbor-Deserts	0	0	1	
*Commercial*				40
Geraldton Sandplains	10	1	3	
Swan Coastal Plain	16	0	13	
Jarrah Forest	20	0	10	
Warren	8	2	6	
Avon Wheatbelt-Yalgoo	6	2	5	
Mallee	7	2	2	
Esperance	6	2	8	
Murchison-Coolgardie	8	2	3	
Northwest-Nullarbor-Deserts	0	0	0

**Table 9:** Regional recovery after site and surrounding burnt, expected number of years until use site again, and for a 50 and 100% return to production, by beekeeper category [Q87].

	Impact of fire^a^					
	Observations	Mean	Std Dev.	25th PC	50th PC	75th PC
*Hobbyist-Amateur*						
Geraldton Sandplains						
Use site again (yrs)	1	10		10	10	10
Return to 50% production (yrs)	1	6		6	6	6
Return to 100% production (yrs)	1	10		10	10	10
Swan Coastal Plain						
Use site again (yrs)	10	6	3.9	2	6	10
Return to 50% production (yrs)	9	4	3.4	1	3	5
Return to 100% production (yrs)	8	11	9.1	3	9	20
Jarrah Forest						
Use site again (yrs)	17	2	1.5	1	2	3
Return to 50% production (yrs)	20	3	5.5	1	2	3
Return to 100% production (yrs)	20	6	8.4	2	3	5
Warren						
Use site again (yrs)	4	3	1.6	2	3	4
Return to 50% production (yrs)	4	1	0.5	1	1	2
Return to 100% production (yrs)	3	3	2.1	1	2	5
Avon Wheatbelt-Yalgoo						
Use site again (yrs)	1	2		2	2	2
Return to 50% production (yrs)	1	1		1	1	1
Return to 100% production (yrs)	1	7		7	7	7
Mallee						
Use site again (yrs)	0					
Return to 50% production (yrs)	0					
Return to 100% production (yrs)	0					
Esperance						
Use site again (yrs)	2	3	2.8	1	3	5
Return to 50% production (yrs)	2	3	2.8	1	3	5
Return to 100% production (yrs)	2	5	3.5	2	5	7
Murchison-Coolgardie						
Use site again (yrs)	0					
Return to 50% production (yrs)	0					
Return to 100% production (yrs)	0					
Northwest-Nullarbor-Deserts						
Use site again (yrs)	0					
Return to 50% production (yrs)	0					
Return to 100% production (yrs)	0					
*Commercial*						
Geraldton Sandplains						
Use site again (yrs)	9	8	3.9	5	7	10
Return to 50% production (yrs)	10	8	4.8	5	6	10
Return to 100% production (yrs)	9	20	14.8	8	15	30
Swan Coastal Plain						
Use site again (yrs)	15	4	1.6	3	4	5
Return to 50% production (yrs)	15	6	3.5	3	5	7
Return to 100% production (yrs)	15	12	12.0	6	10	19
Jarrah Forest						
Use site again (yrs)	17	4	1.6	3	4	5
Return to 50% production (yrs)	17	6	4.4	4	4	5
Return to 100% production (yrs)	15	13	12.8	6	10	20
Warren						
Use site again (yrs)	5	10	11.4	4	5	5
Return to 50% production (yrs)	6	8	6.9	5	5	6
Return to 100% production (yrs)	5	11	11.0	7	7	7
Avon Wheatbelt-Yalgoo						
Use site again (yrs)	4	6	3.0	4	5	8
Return to 50% production (yrs)	3	6	3.5	3	6	10
Return to 100% production (yrs)	3	12	7.2	6	10	20
Mallee						
Use site again (yrs)	5	12	4.8	10	10	10
Return to 50% production (yrs)	6	12	6.0	8	10	20
Return to 100% production (yrs)	6	37	17.4	21	46	50
Esperance						
Use site again (yrs)	5	15	5.1	10	13	20
Return to 50% production (yrs)	6	14	5.3	10	13	20
Return to 100% production (yrs)	5	29	19.5	15	20	50
Murchison-Coolgardie						
Use site again (yrs)	7	15	11.7	4	10	30
Return to 50% production (yrs)	8	20	17.6	7	13	35
Return to 100% production (yrs)	6	26	19.7	14	18	50
Northwest-Nullarbor-Deserts						
Use site again (yrs)	0					
Return to 50% production (yrs)	0					
Return to 100% production (yrs)	0					

Notes

25th PC i.e. 25th percentile, 25% of values are below this value.

50th PC i.e. 50th percentile, 50% of values are below this value.

75th PC i.e. 75th percentile, 75% of values are below this value.

a Excludes zero values.

**Table 10:** Ever had site and surrounding 3 km logged, by beekeeper category and by region [Q85].

	Logging impacted site			
Response	Yes	Unsure	No	Response total
*Hobbyist-Amateur*				
Jarrah Forest	10	5	73	88
Warren	1	4	30	35
*Commercial*				
Jarrah Forest	15	2	14	31
Warren	9	2	5	16

**Table 11:** Recovery after site and surrounding logged, expected number of years until use site again, production losses and years site impacted, by beekeeper category [Q88].

	Impact of logging^a^					
	Observations	Mean	Std Dev.	25th PC	50th PC	75th PC
*Hobbyist-Amateur*						
Jarrah Forest						
Use site again (yrs)	3	3	2.1	1	2	5
Loss of production (%)	5	30	40.4	3	20	25
Years site impacted (yrs)	6	20	23.5	3	12	30
Warren						
Use site again (yrs)	1	30		30	30	30
Loss of production (%)	1	100		100	100	100
Years site impacted (yrs)	1	30		30	30	30
						
*Commercial*						
Jarrah Forest						
Use site again (yrs)	11	24	31.1	2	7	40
Loss of production (%)	10	62	39.9	20	67	100
Years site impacted (yrs)	10	27	39.3	4	6	30
Warren						
Use site again (yrs)	8	36	32.1	11	33	51
Loss of production (%)	7	66	39.1	20	80	100
Years site impacted (yrs)	7	39	44.0	3	20	100

Notes

25th PC i.e. 25th percentile, 25% of values are below this value.

50th PC i.e. 50th percentile, 50% of values are below this value.

75th PC i.e. 75th percentile, 75% of values are below this value.

a Excludes zero values.

### 1.7 Honey Sales and Distribution (Tables 12 and 13)

**Table 12:** Volume of honey sold or distributed in season July 2019–June 2020, by flora type and beekeeper category [Q102].

	Honey volume (kg/yr)					
Honey flora type	Observations^a^	Mean	Std Dev.	25th PC	50th PC	75th PC
*Backyard beekeepers*						
Mixed flora	77	24	21.9	10	20	30
Mono flora - not TA tested	6	27	13.8	15	25	35
Organic mixed flora	5	16	10.5	10	15	22
Organic mono flora - not TA tested	2	55	63.6	10	55	100
TA 10+ - tested	0					
TA 20+ - tested	0					
TA 30+ or more - tested	0					
Organic TA 10+ - tested	0					
Organic TA 20+ - tested	0					
Organic TA 30+ or more - tested	0					
Manuka - Leptospermum	0					
**Response total**	**87**					
*Hobbyist-Amateur beekeepers*						
Mixed flora	175	135	229.2	20	60	150
Mono flora - not TA tested	34	83	70.5	30	60	100
Organic mixed flora	11	72	89.8	15	35	135
Organic mono flora - not TA tested	5	211	273.4	10	45	400
TA 10+ - tested	0					
TA 20+ - tested	2	26	34.6	1	26	50
TA 30+ or more - tested	2	190	240.4	20	190	360
Organic TA 10+ - tested	0					
Organic TA 20+ - tested	0					
Organic TA 30+ or more – tested^b^	n/a					
Manuka - Leptospermum	0					
**Response total**	**196**					
*Commercial beekeepers*						
Mixed flora	31	4,496	6,865.5	320	1,500	4,225
Mono flora - not TA tested	21	12,194	20,038.9	620	2,500	17,000
Organic mixed flora	3	70,667	103,736.8	2,000	20,000	190,000
Organic mono flora - not TA tested	2	3,500	2,121.3	2,000	3,500	5,000
TA 10+ - tested	0					
TA 20+ - tested	2	6,050	8,414.6	100	6,050	12,000
TA 30+ or more - tested	5	6,740	5,868.4	1,700	6,000	10,000
Organic TA 10+ - tested	0					
Organic TA 20+ - tested^b^	n/a					
Organic TA 30+ or more - tested	3	10,333	8,386.5	5,000	6,000	20,000
Manuka - Leptospermum	2	3,500	2,828.4	1,500	3,500	5,500
**Response total**	**31**					

Notes

25th PC i.e. 25th percentile, 25% of values are below this value.

50th PC i.e. 50th percentile, 50% of values are below this value.

75th PC i.e. 75th percentile, 75% of values are below this value.

Response total is the number of respondents who answered the question.

a Excludes respondents whose values were less than 1.

b - n/a - Not available for publication, too few observations.

**Table 13:** Price of honey sold or distributed in season July 2019–June 2020, by flora type and beekeeper category [Q103].

	Honey price ($/kg)					
Honey flora type	Observations^a^	Mean	Std Dev.	25th PC	50th PC	75th PC
*Backyard beekeepers*						
Mixed flora	39	13.1	5.62	10.0	13.0	16.0
Mono flora - not TA tested	2	10.0	7.07	5.0	10.0	15.0
Organic mixed flora	2	14.5	0.71	14.0	14.5	15.0
Organic mono flora - not TA tested	2	12.0	2.83	10.0	12.0	14.0
TA 10+ - tested	0					
TA 20+ - tested	0					
TA 30+ or more - tested	0					
Organic TA 10+ - tested	0					
Organic TA 20+ - tested	0					
Organic TA 30+ or more - tested	0					
Manuka - Leptospermum	0					
**Response total**	**43**					
*Hobbyist-Amateur beekeepers*						
Mixed flora	141	13.4	5.40	10.0	13.0	15.0
Mono flora - not TA tested	29	13.9	5.00	10.0	15.0	16.0
Organic mixed flora	9	17.8	6.67	15.0	15.0	20.0
Organic mono flora - not TA tested	3	13.7	6.99	6.2	15.0	20.0
TA 10+ - tested	0					
TA 20+ - tested	1	25.0		25.0	25.0	25.0
TA 30+ or more - tested^b^	n/a					
Organic TA 10+ - tested	0					
Organic TA 20+ - tested	0					
Organic TA 30+ or more – tested	1	20.0		20.0	20.0	20.0
Manuka - Leptospermum	0					
**Response total**	**159**					
*Commercial beekeepers*						
Mixed flora	30	10.1	5.38	6.0	9.3	14.0
Mono flora - not TA tested	20	10.3	5.88	6.0	8.5	13.0
Organic mixed flora	3	8.3	3.21	6.0	7.0	12.0
Organic mono flora - not TA tested	2	11.0	7.07	6.0	11.0	16.0
TA 10+ - tested	0					
TA 20+ - tested^b^	n/a					
TA 30+ or more - tested	4	38.8	11.09	30.0	40.0	47.5
Organic TA 10+ - tested	0					
Organic TA 20+ - tested	1	18.0		18.0	18.0	18.0
Organic TA 30+ or more - tested	2	30.0	7.07	25.0	30.0	35.0
Manuka - Leptospermum	2	13.5	9.19	7.0	13.5	20.0
**Response total**	**31**					

Notes

25th PC i.e. 25th percentile, 25% of values are below this value.

50th PC i.e. 50th percentile, 50% of values are below this value.

75th PC i.e. 75th percentile, 75% of values are below this value.

Response total is the number of respondents who answered the question.

a Excludes respondents whose values were less than or equal to 1.

b - n/a - Not available for publication, too few observations.

### 1.8 Operating Costs (Table 14)

**Table 14:** Operating costs per hive and per kilogram of honey, by beekeeper category in season July 2019–June 2020 [Q113].

	Total cost ($/unit)					
Unit cost	Observations	Mean	Std Dev.	25th PC	50th PC	75th PC
*Hobbyist-Amateur*						
Cost per hive ($/hive)^a^	36	171.0	139.64	75.0	136.2	224.3
Cost per kilogram of honey ($/kg)^b^	35	14.2	12.97	4.0	10.1	22.0
*Commercial*						
Cost per hive ($/hive)^a^	23	306.1	257.66	60.0	245.0	628.3
Cost per kilogram of honey ($/kg)^b^	22	12.7	14.60	3.8	7.1	16.8

Notes

25th PC i.e. 25th percentile, 25% of values are below this value.

50th PC i.e. 50th percentile, 50% of values are below this value.

75th PC i.e. 75th percentile, 75% of values are below this value.

a Excludes values > 800.

b Excludes values > 100.

### 1.9 Pollination Services to Agricultural Crops, Fees and Future Pollinators (Table 15, Table 16, Table 17)

**Table 15:** Beekeepers pollinating crops, season July 2019-June 2020 [Q60].

Crop	Observations	Response total
Avocado	13	
Canola	3	
Citrus, including orange, mandarin, grapefruit, lemons and limes	3	
Gourds, including cucumber, squash, zucchini, pumpkin	1	
Nuts, excluding almond	2	
Pome fruit, including apple, pear, nashi	2	
Stone fruit, including apricot, cherry, nectarine, peach, mango	6	
		20

**Table 16:** Pollination hive numbers and service fee, season July 2019–June 2020 [Q58, Q63].

Response	Observations	Mean	Std Dev.	25th PC	50th PC	75th PC
Number of hives^a^	16	139	170.6	21	80	193
Pollination service fee ($/hive)^b^	13	167.69	41.312	150	168	200

Notes

25th PC i.e. 25th percentile, 25% of values are below this value.

50th PC i.e. 50th percentile, 50% of values are below this value.

75th PC i.e. 75th percentile, 75% of values are below this value.

a Excludes values < 5.

b Excludes values < 10.

**Table 17:** Beekeepers considering offering pollination services in next 5 years [Q175].

	Beekeeper category			
Response	Backyard	Hobbyist-Amateur	Commercial	Total
Yes	4	16	12	32
Maybe	12	31	6	49
No	146	197	7	350
**Response total**	**162**	**244**	**25**	**431**

## 2 Experimental Design, Materials and Methods

The survey design was developed through semi-structured interviews with beekeepers and industry representatives to gain an understanding of the use of natural resources, the production system and challenges facing the industry with respect to apiary sites, bush fires and logging. This was followed by an extensive literature review of surveys and questionnaires conducted within the apiculture industry and on related topics. Manning's [2] Natural Resource Questionnaire for Beekeepers targeted amateur and commercial beekeepers in Western Australia in 1990-91 and focused on honey production and geographical significance of apiary sites. This previous work was valuable and influenced the construction of the online survey to be inclusive of all beekeepers and registered apiary site holders in Western Australia. An apiculture monitoring programme in New Zealand that publishes annual reports and survey data was also reviewed [3].

The Natural Resources for Beekeepers Questionnaire (Western Australia) 2020-21 [4] was developed using Qualtrics software [5] using a range of question types such as multiple choice, multiple answer with graphics, number selection using slider bars and free text entry. Side-by-side type questions were best suited for displaying calendar months to ascertain region use throughout the year. Often selected choices were carried forward to gather more information about specific topics, for example the crops selected for pollination were later redisplayed in questions asking for the regions of these crops, hive stocking rate, crop area and service fee charged. To avoid unnecessary time spent on irrelevant questions, survey flow and question display logic were carefully constructed to generate a question pathway for respondents that was applicable to them and their situation.

The questionnaire accommodated all scales of production and use of natural resources. In total there were 180 questions, but the questions seen by the participant varied depending on previous responses. Statements and information sections such as regional maps, descriptive displays and links to more information were included throughout the questionnaire for clarity. The questions mostly focused on the season July 2019–June 2020, and there were options to provide historical information if respondents wanted to. Crop pollinators were included and retired full time/commercial beekeepers who had retired in the last ten years had the option to share their valuable knowledge and experience from their years of commercial beekeeping.

The beekeepers were concerned with privacy and sharing commercially sensitive knowledge via a survey. Therefore, to protect anonymity random sampling from the population was not possible, instead the sample was self-selected by those willing to participate in the survey. The online survey was open to all Western Australian beekeepers and registered apiary site licence holders during December 2020 to June 2021, and participation was voluntary and anonymous. Invitation to participate was delivered through industry networks and newsletters, websites, Facebook and direct email. All participants had the option to seek assistance if required.

Statistical analysis of survey results including data clean up, table and graph creation has been performed using Stata/SE 16.1 [6] and Microsoft Excel. Beekeepers were classified according to the number of hives, number and type of sites and honey harvested. Beekeeper categories were analyzed separately, and no all-encompassing single conclusion was attempted.

## Ethics Statements

This research was conducted under the University of Western Australia ethics protocol RA/4/20/6491. All research participants provided informed consent.

## CRediT authorship contribution statement

**Cheryl Day:** Conceptualization, Methodology, Software, Validation, Formal analysis, Investigation, Resources, Data curation, Writing – original draft, Writing – review & editing, Visualization, Project administration. **Benedict White:** Conceptualization, Methodology, Formal analysis, Writing – review & editing, Supervision, Project administration, Funding acquisition.

## Declaration of Competing Interest

The authors declare that they have no known competing financial interests or personal relationships that could have appeared to influence the work reported in this paper.

## Data Availability

A survey dataset to better understand the honey bee industry, use and value of natural resources and challenges for beekeepers in Western Australia: A beekeepers' perspective (Original data) (Pure, The University of Western Australia Research Repository).
